# Urachal Remnant Calcification: A Rare Cause of Calcification Within the Urinary Bladder

**DOI:** 10.7759/cureus.29443

**Published:** 2022-09-22

**Authors:** Nil Rawal, Pierre Maldjian

**Affiliations:** 1 Radiology, Rutgers University, Newark, USA

**Keywords:** median umbilical ligament calcification, bladder, median umbilical ligament, urachal remnant, bladder calcification

## Abstract

Calcification of the urachal remnant is a rare cause of urinary bladder calcification. We present a case of calcification of the urachal remnant found incidentally on computed tomography (CT) scan in the setting of trauma. Our case clearly illustrates that reformatting images in the sagittal plane can clearly delineate the median umbilical ligament and its relationship to the calcification for confirmation of the diagnosis. Recognition of the characteristic appearance of this entity ensures prompt diagnosis and avoids unnecessary workup for other causes of calcification within the bladder.

## Introduction

Urinary bladder calcifications can be secondary to calculi, infection, neoplasm, chemotherapy, or radiation treatment [1]. Urachal remnant calcification is a rare entity with only nine reported cases. These include a single case described by Rodrigues and a series of eight cases described by Parthasarathi et al. [2,3]. The calcification is characteristically located at the anterior wall of the bladder, at the insertion of the embryologic urachus. The urachus is a fetal tubular conduit that drains urine from the bladder to the umbilicus during the first trimester and obliterates at the 12th week of gestation into a fibrous cord known as the median umbilical ligament. The mechanism of calcification is likely similar to dystrophic calcification of other fetal structures that have undergone fibrous degeneration, such as the ligamentum arteriosum [4]. Here we present a case of calcification of the urachal remnant, an unusual cause of calcification in the urinary bladder, found incidentally on computed tomography (CT). We illustrate that images reformatted in the sagittal plane clearly discriminate the location of calcification to the attachment of the median umbilical ligament along the anterior wall of the bladder. Recognition of the typical appearance of this benign finding ensures prompt diagnosis and avoids unnecessary workup for other etiologies of urinary bladder calcification. In this report, we discuss the appearance of this entity on CT, the classification of urachal anomalies, and the differential diagnosis of urinary bladder calcification. 

## Case presentation

A 44-year-old man presented to the emergency department after a motor vehicle accident. He complained of chest wall and abdominal pain. On physical exam, he had tenderness over his left chest wall and abdomen. Vital signs and laboratory findings were normal. He had no pertinent past medical history. A CT scan of the abdomen and pelvis with intravenous contrast (no oral or rectal contrast) was performed to evaluate for internal injuries.

**Figure 1:**
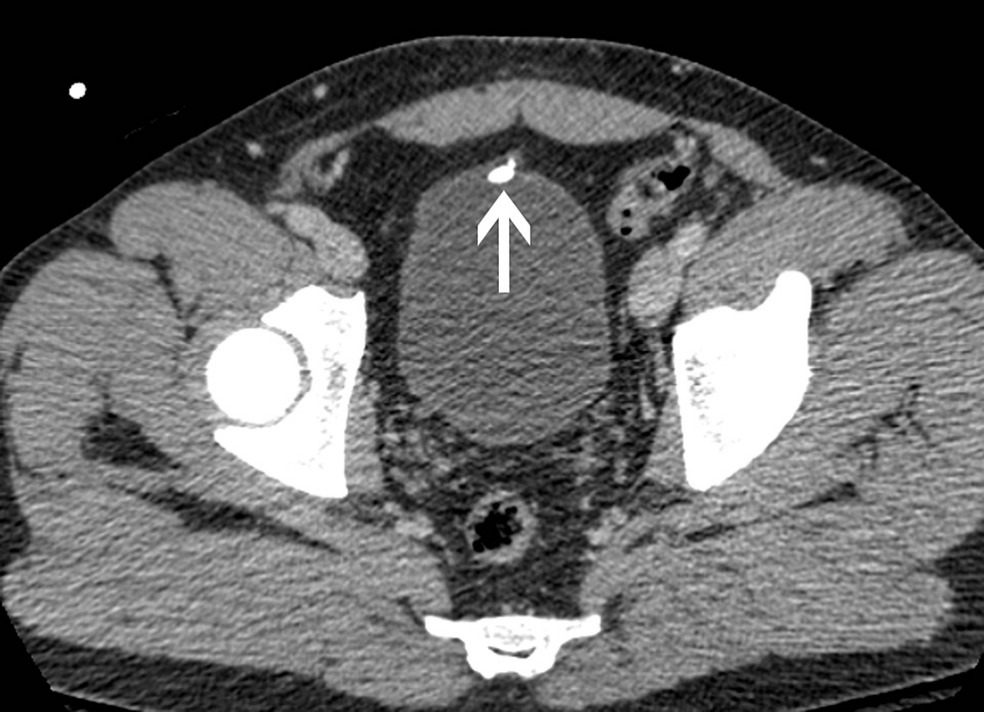
Axial CT image throughout the pelvis shows a 7 mm calcification (arrow) attached to the midline of the anterior wall of the urinary bladder.

**Figure 2:**
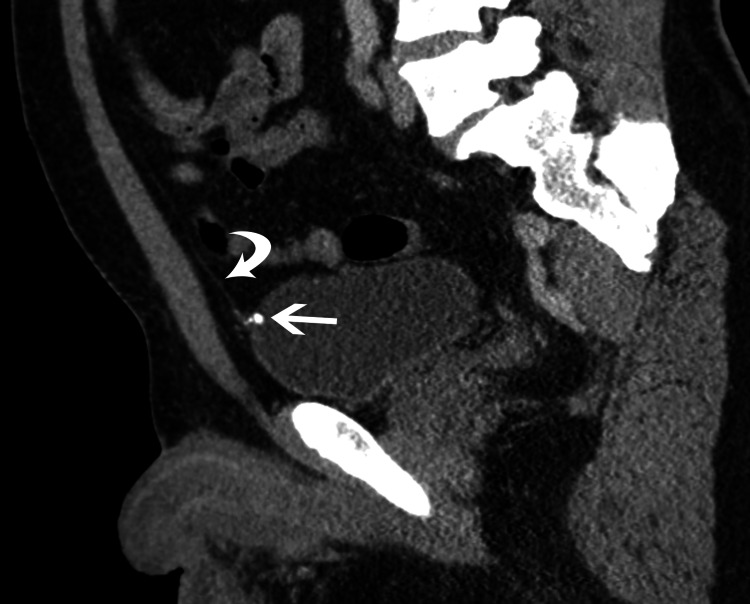
Sagittal reformatted image from CT scan shows cord-like midline structure (curved arrow) extending from the bladder toward the umbilicus representing the median umbilical ligament. The calcifications (straight arrow) are located at the insertion of the ligament onto the urinary bladder. Note that there is no outpouching of the bladder wall at the insertion of the ligament to suggest the formation of a urachal diverticulum.

No traumatic injuries were present but the CT study revealed nondependent calcifications, the largest measuring 7 mm, attached to the anterior inner wall of the urinary bladder (Figure 1). Sagittal reformatted views showed that the calcifications were located at the bladder attachment of the median umbilical ligament (Figure 2). The incidental diagnosis of calcification of the urachal remnant was made and the patient was discharged from the emergency room.

## Discussion

The urachus is an embryonic remnant of the allantois and cloaca that during fetal life originates at the urinary bladder and extends as a tubular tract to the umbilicus. Typically, before birth, the urachus obliterates to form a fibrous cord-like structure called the median umbilical ligament. The fibrous remnant lies extraperitoneal in the space of Retzius, posterior to the transverse fascia and anterior to the peritoneum. Rarely, with an incidence of 1 in 5000 births, the urachus persists as a congenital anomaly categorized as one of the following: patent urachus, urachal cyst, urachal diverticulum or urachal sinus. A patent urachus, the most common anomaly, is a fluid-filled communication between the umbilicus and the bladder. A urachal cyst, the second-most common anomaly, is a fluid-filled dilation of the mid-urachus. A urachal diverticulum is a blind focal dilation at the bladder end of the urachus while a urachal sinus is a blind focal dilation at the umbilical end of the urachus [5-10]. In our case, a 7 mm calcific density was present on the inner surface of the anterior wall of the bladder, midline at the insertion of the urachus. While the precise embryologic explanation for this finding has not been described, we speculate that it may result from dystrophic calcification within a small amount of persistent urachal tissue. While the true incidence of urachal remnant calcification is difficult to determine due to the paucity of literature on the subject, one single-center case series demonstrated an incidence of 1.3% (8 out of 600 cases) in patients who received CT scans for symptoms consistent with renal colic [3]. This entity is benign and does not require further workup.

The differential diagnosis for calcification within the urinary bladder or bladder wall includes bladder stone, neoplasm such as adenocarcinoma or transitional cell carcinoma, and infection especially from schistosomiasis and tuberculosis [1]. Calcification of the urachal remnant can be easily mistaken for a bladder stone on CT especially if the patient is imaged in the prone position [2,3,11]. On CT, a bladder stone, due to its mobility, appears as a calcification along the gravitationally dependent surface of the bladder. It can be associated with bladder wall thickening or bladder diverticula [12,13]. Patients suspected of having acute renal colic from a ureteral stone may undergo CT in the prone position to differentiate a small stone impacted in the ureterovesical junction (which would appear as a calcification along the posterior bladder surface at the ureteral insertion) from a recently passed stone (which would appear as a small calcification along the gravitationally dependent anterior surface of the bladder in the prone position) [13]. Furthermore, the radiologist should also consider the possibility of a urachal cyst or vesicourachal diverticulum containing a calculus [7, 11]. Unlike our case, these entities appear as fluid-filled sac-like structures protruding outward from the bladder wall.

Bladder cancers may calcify but typically present as soft tissue attenuation bladder masses on CT. Neoplasm, most commonly adenocarcinoma, can arise in persistent urachal tissue. Urachal carcinoma presents as a soft tissue mass at the midline near the dome of the bladder or anywhere along the course of the urachus (from the bladder to the umbilicus). These tumours are usually 5-6 cm in size with a mixed cystic-solid appearance and a prominent extravesical component. On CT, 70% of urachal carcinomas contain calcification usually along the periphery [14]. Bladder transitional cell carcinoma, the most common type of bladder neoplasm, can occasionally calcify and usually appears as focal thickening or mass within the bladder wall at any location [15].

Schistosomiasis is the most common infectious cause of bladder calcification. On CT schistosomiasis is characterized by a fibrotic thick-walled bladder with curvilinear, thin arcuate calcifications along the bladder wall which represent calcified parasitic eggs [1,16]. Tuberculosis can also cause calcification of the bladder. CT findings include a contracted bladder with calcifications and scarring involving the kidneys and bladder simultaneously [1,17]. Chronic cystitis from radiation or intravesical chemotherapy may also result in calcification within a fibrosed contracted bladder but is usually apparent from a review of the patient’s medical history [1,18].

## Conclusions

Calcification of the urachal remnant occurs at the insertion of the median umbilical ligament on the anterior wall of the bladder and has a characteristic appearance on CT. It should not be mistaken for a bladder stone or other cause of bladder calcification. The significance of our case is that it clearly illustrates that reformatting images in the sagittal plane can clearly delineate the median umbilical ligament and its relationship to the calcification for confirmation of the diagnosis.
